# IS COMBINED TRAINING MORE EFFECTIVE THAN SINGLE-DOMAIN TRAINING: A RANDOMIZED CONTROLLED TRIAL WITH OLDER ADULTS

**DOI:** 10.1093/geroni/igz038.2644

**Published:** 2019-11-08

**Authors:** Soledad Ballesteros, Jennifer Rieker, josé M Reales, julia Mayas, María Pilar Jiménez, Antonio Prieto, Pilar Toril

**Affiliations:** 1 Universidad Nacional de Educación a Distancia, Madrid, Spain, Spain; 2 Centro Cardenal Cisneros (Alcalá de Henares), Madrid (Spain), Spain

## Abstract

Previous research suggests that both cognitive training and physical exercise help to maintain brain health and cognitive functions that decline with age. The main objectives of this four-arms RCT are (1) to investigate the synergetic effects of a group-based multidomain training program that combines cognitive video-game training with physical exercise, in comparison to those produced by cognitive training combined with physical control activity, physical training combined with cognitive control activity, or a combination of both control activities; (2) to investigate in a memory-based task switching task whether event Related Potential (ERP) latencies of the P2 component are shorter, and N2 and P3b components are enhanced after training; and (3) to find out whether possible enhancements persist after a 3-month period without training. One hundred and twenty participants will be randomly assigned to one of the four combinations of cognitive training and physical exercise. The cognitive component will be either video-game training (cognitive intervention, CI) or video games not specifically designed to train cognition (cognitive control, CC). The physical exercise component will either emphasize endurance, strength, and music-movement coordination (exercise intervention, EI) or stretching, toning and relaxation (exercise control, EC). This RCT will investigate the short and long-term effects of combined multi-domain training compared to cognitive training and physical training alone, on executive control and memory functions of healthy older adults, in comparison with the performance of an active control group. This trial is an ongoing project started in 2018. Trial registration: Clinicaltrials.gov ID: NCT03823183; https://register.clinicaltrials.gov/

